# CCAR1 promotes chromatin loading of androgen receptor (AR) transcription complex by stabilizing the association between AR and GATA2

**DOI:** 10.1093/nar/gkt644

**Published:** 2013-07-25

**Authors:** Woo-Young Seo, Byong Chang Jeong, Eun Ji Yu, Hwa Jin Kim, Seok-Hyung Kim, Joung Eun Lim, Ghee Young Kwon, Hyun Moo Lee, Jeong Hoon Kim

**Affiliations:** ^1^Department of Health Sciences and Technology, Samsung Advanced Institute for Health Sciences and Technology, Sungkyunkwan University, Seoul, 135-710, Korea, ^2^Department of Biomedical Sciences, Samsung Biomedical Research Institute, Samsung Medical Center, Seoul, 135-710, Korea, ^3^Department of Urology, Samsung Medical Center, Sungkyunkwan University School of Medicine, Seoul, 135-710, Korea and ^4^Department of Pathology, Samsung Medical Center, Sungkyunkwan University School of Medicine, Seoul, 135-710, Korea

## Abstract

Androgen receptor (AR), a ligand-dependent transcription factor, plays a critical role in prostate cancer onset and progression, and its transcriptional function is mediated largely by distinct nuclear receptor co-regulators. Here, we show that cell cycle and apoptosis regulator 1 (CCAR1) functions as an AR co-activator. CCAR1 interacted with and enhanced the transcriptional activity of AR. Depletion of CCAR1 caused reduction in androgen-dependent expression of a subset of AR target genes. We further showed that CCAR1 is required for recruitment of AR, MED1 and RNA polymerase II to the enhancers of AR target genes and for androgen-induced long-range prostate specific antigen enhancer–promoter interaction. The molecular mechanism underlying CCAR1 function in AR-mediated transcription involves CCAR1-mediated enhanced recruitment of GATA2, a pioneer factor for AR, to AR-binding sites. CCAR1 stabilized the interaction between AR and GATA2 by interacting directly with both proteins, thereby facilitating AR and GATA2 occupancy on the enhancers. Furthermore, CCAR1 depletion inhibited the growth, migration, invasion of prostate cancer cells and reduced the tumorigenicity of prostate cancer cells *in vivo*. Our results firmly established CCAR1 as an AR co-activator that plays a key role in AR transcription complex assembly and has an important physiological role in androgen signaling and prostate tumorigenesis.

## INTRODUCTION

Androgen receptor (AR), a member of the nuclear receptor (NR) superfamily, functions as a ligand-dependent transcription factor that plays a key role in the growth and maintenance of the normal prostate and the onset and progression of prostate cancer (1,2). Transcriptional activation by AR and other NRs is tightly controlled by a complex process, including DNA sequence, epigenetic signatures, chromatin structure and dynamic interactions between NRs and gene specific co-activators at the target enhancers and promoters (3,4). On activation, AR binds to androgen response elements (AREs) in the enhancer and promoter regions of target genes. Recent genome-wide ChIP-on-chip studies have shown that motifs for pioneer factors (e.g. FoxA1, GATA2 and Oct1) are significantly enriched within the AR-binding regions, compared with genomic background (5,6). The pioneer factors FoxA1 and GATA2 are recruited to a significant portion of AR-binding sites in prostate cancer cells, facilitating AR binding to AREs by interacting directly with AR and consequently enhancing AR-mediated transcription. Once bound to AREs, AR regulates the expression of a variety of target genes through the recruitment of co-regulators (co-activators and co-repressors) that mediate local chromatin remodeling as well as communications with the RNA polymerase II (Pol II)-associated basal transcription machinery (4,5,7). Although there has been considerable progress in describing the roles of AR co-regulators in AR-mediated gene regulation, little is known about their roles in the interplay between AR and pioneer factors.

Cell cycle and apoptosis regulator 1 (CCAR1) is a regulator of apoptosis signaling as well as cell proliferation. Recently, we have shown that CCAR1 interacts with estrogen receptor α (ERα) and cooperates synergistically with components of the p160 co-activator complex (8). In addition, CCAR1 associates with components of the Mediator complex and facilitates recruitment of Mediator complex to the promoter of target genes by providing a physical link between p160 co-activator and Mediator complexes. CCAR1 is important for estrogen-induced expression of ERα target genes and estrogen-dependent growth of breast cancer cells. Deletion in breast cancer (DBC1), which shows significant homology to CCAR1, has been reported as an AR co-activator (9). DBC1 interacts with AR and enhances AR-mediated transcription by promoting DNA-binding activity of AR. Recently, we identified DBC1 as a CCAR1-binding protein and showed that DBC1 associates directly with ERα and cooperates synergistically with CCAR1 to enhance ERα and other NRs function (10). To further characterize the mechanism by which CCAR1 contributes to transcriptional activation by NRs, we explored the possibility that CCAR1 may play a role in AR-mediated transcription and contribute to the tumorigenic potential of prostate cancer cells.

## MATERIALS AND METHODS

### Cell culture and transient transfection

LNCaP cells were maintained in RPMI 1640 supplemented with 10% fetal bovine serum (FBS). CV-1, 293T and VCaP cells were cultured in Dulbecco’s modified Eagle’s medium with 10% FBS. To generate LNCaP cells stably expressing luciferase (LNCaP-LUC), LNCaP cells were infected with lentiviruses expressing FLAG-tagged luciferase and selected with hygromycin. For chromatin immunoprecipitation (ChIP), RNA interference (RNAi) and methylthiazol tetrazolium (MTT) assays, LNCaP cells were grown in RPMI supplemented with 5% dextran/charcoal-stripped FBS. Transient transfections and reporter gene assays were performed as described previously (8,10). Each experiment was repeated independently at least three times. The results shown are the means and SD of triplicate points.

### ChIP assays

LNCaP cells were grown in RPMI1640 supplemented with 5% dextran/charcoal-stripped serum in 150-mm dishes for 3 days and then treated with or without 10 nM dihydrotestosterone (DHT) for 16 h. ChIP experiments were performed according to the procedure described previously (8,11). The immunoprecipitated DNAs were amplified by qPCR. The antibodies and primers used are listed in Supplementary Materials and Methods.

### RNAi and real time quantitative reverse transcriptase-PCR

The depletion of CCAR1 was performed according to previously described protocol (8). LNCaP and VCaP cells were infected with a lentivirus encoding a non-specific (NS) short hairpin RNA (shRNA) or CCAR1 shRNA using Polybrene (Millipore). After infection, cells were selected with 2 µg/ml puromycin. Quantitative real-time reverse transcriptase-PCR (qRT-PCR) was performed with total RNA and Brilliant SYBR Green QRT-PCR Master Mix 1-Step (Stratagene). The primers used are listed in Supplementary Materials and Methods.

### Chromosome conformation capture assays

Chromosome conformation capture (3C) assays were performed according to the procedure described previously with some modifications (7,12). The cross-linked chromatin was digested overnight with 200U of BstYI, and the restriction enzyme was heat inactivated. The digested chromatin was diluted 50-fold in ligase buffer and ligated overnight with T4 DNA ligase. After reverse crosslinking, DNA was extracted with phenol/chloroform and precipitated with ethanol. The 3C DNAs were amplified by PCR. The primers used are listed in Supplementary Materials and Methods.

### Formaldehyde-assisted isolation of regulatory elements assays

The procedure for formaldehyde-assisted isolation of regulatory elements (FAIRE) was performed as described previously (13). Briefly, chromatin was crosslinked with 1% formaldehyde and sheared by sonication. Sheared chromatin was subjected to phenol/chloroform extraction to recover DNA not bound by nucleosome in the water phase. The samples were reverse crosslinked, and the isolated DNAs were purified. The relative enrichment of open chromatin for prostate specific antigen (PSA) and TMPRSS2 enhancer regions was quantified by qPCR using ChIP PCR primers for the PSA and TMPRSS2 enhancers.

Plasmids and antibodies, protein interaction assays and immunoblot, gene expression analysis by Affymetrix microarray, MTT, migration, invasion, colony formation assays, xenograft experiments, immunohistochemical staining, DNA affinity precipitation (DAPA) assays and statistical analysis are explained in detail in Supplementary Data.

## RESULTS

### CCAR1 functions as an AR co-activator

**Figure 1.**
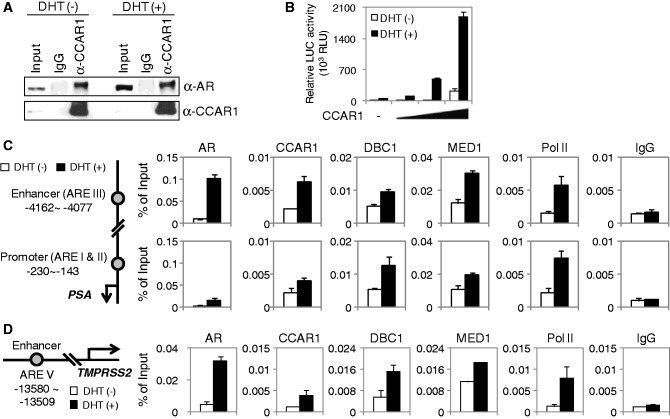
CCAR1 interacts with AR and functions as an AR co-activator. (**A**) Endogenous interaction between CCAR1 and AR. LNCaP cells were treated with ethanol or 10 nM DHT for 16 h. Cell lysates were prepared and immunoprecipitated with normal IgG or anti-CCAR1. The immunoprecipitates were analyzed by immunoblot with the indicated antibodies. (**B**) CV-1 cells were transfected with pSG5.HA-AR (10 ng) and MMTV-LUC reporter (200 ng) in combination with various amounts (200, 400 and 800 ng) of pSG5.HA-CCAR1 and grown in medium containing or lacking 20 nM DHT before conducting luciferase assays on cell extracts. Data are means ± SD (n = 3). (**C** and **D**) Recruitment of CCAR1 to AR target genes. Cross-linked, sheared chromatin from LNCaP cells treated with or without 10 nM DHT (16 h) was immunoprecipitated with the indicated antibodies. qPCR analyses were performed using primers specific for the PSA enhancer and promoter (C) and TMPRSS2 enhancer (D). The results are shown as percentage of input and are means ± SD (n = 3).

Given our findings that CCAR1 interacts with members of the NR family (8), such as ERα and glucocorticoid receptor (GR), we first examined the association between endogenous CCAR1 and AR in AR-positive LNCaP prostate cancer cells by coimmunoprecipitation (CoIP) assays. CCAR1 bound to AR in a DHT-independent manner (Figure 1A). Similarly, HA-tagged CCAR1 was coimmunoprecipitated specifically with FLAG-tagged AR from extracts of transiently transfected 293T cells (Supplementary Figure S1A). In addition, *in vitro* glutathione-S-transferase (GST) pull-down assays confirmed the interaction between CCAR1 and AR (Supplementary Figure S1B) and showed that CCAR1 interacts with the C-terminal region of AR in a hormone-independent manner (Supplementary Figure S1C, D and E), suggesting that CCAR1 interacts directly with AR. We next tested whether CCAR1 can function as a co-activator for AR. In reporter gene assays, CCAR1 enhanced the hormone-induced AR activity in a dose-dependent manner (Figure 1B), and coexpression of CCAR1 and DBC1 enhanced AR function in a synergistic manner (Supplementary Figure S2). These results suggest that CCAR1 functions as an AR co-activator and cooperates synergistically with DBC1.

To examine whether CCAR1 is directly involved in AR-mediated transcription, we performed ChIP assays in prostate cancer cells. Previous studies have shown that AR, MED1/TRAP220 (a subunit of the Mediator complex) and RNA Pol II are recruited to the enhancer (ARE III located 4.2 kb upstream of transcription start site) and promoter (ARE I and II located 170 and 400 bp upstream of transcription start site) of PSA (also termed KLK3 for kallikrein-related peptidase 3) gene (7,14,15). As shown in Figure 1C, AR, MED1, RNA Pol II and DBC1 were recruited to the PSA enhancer and promoter in a hormone-dependent manner, as reported previously (7,9,14,15). DHT treatment also led to the recruitment of CCAR1 to the PSA enhancer and promoter in LNCaP and VCaP cells, whereas the normal IgG control was not affected by DHT (Figure 1C and Supplementary Figure S3A). A similar recruitment pattern of these proteins was observed on the enhancer of another AR target gene, TMPRSS2 (Figure 1D and Supplementary Figure S3B), indicating that CCAR1 is recruited to AR binding sites in AR target genes and is directly involved in the transcriptional regulation of endogenous AR target genes.

### CCAR1 is required for DHT-induced expression of a subset of AR target genes

**Figure 2.**
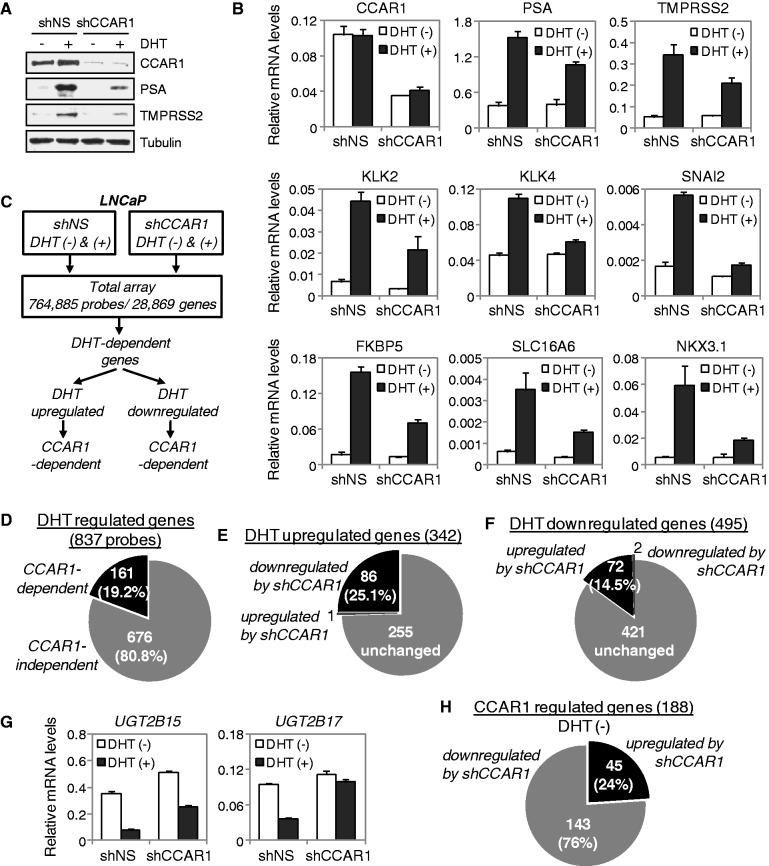
Requirement of CCAR1 for AR function and identification of CCAR1-dependent target genes by microarray analysis. (**A** and **B**) LNCaP cells infected with lentiviruses encoding a non-specific (shNS) or CCAR1 shRNA (shCCAR1) were treated with 10 nM DHT or ethanol vehicle for 24 h. Protein levels were monitored by immunoblot using the indicated antibodies (A). Total RNA was examined by real-time qRT-PCR analysis with primers specific for the indicated mRNAs (B). Results shown were normalized to β-actin mRNA levels and are means ± SD (n = 3). (**C**) Flow chart depicting the strategy of cDNA microarray analysis and CCAR1-dependent gene selection process. (**D**) CCAR1-dependent genes represent 19.2% of all differentially expressed genes by DHT. (**E**) Pie graph shows that among all DHT-upregulated genes, 25.1% of them are downregulated by CCAR1 depletion. (**F**) Pie graph shows that among all DHT-downregulated genes, 14.5% of them are upregulated by CCAR1 depletion. (**G**) CCAR1 is required for androgen-repressed expression of UGT2B15 and UGT2B17 genes. LNCaP cells expressing shNS or shCCAR1 were treated with 10 nM DHT or ethanol vehicle for 24 h. The indicated mRNA levels were determined by qRT-PCR as described in (B). (**H**) Pie graph shows that 188 genes are differentially expressed by CCAR1 depletion in the absence of DHT.

To assess the functional involvement of CCAR1 in AR-mediated transcription, the expression of CCAR1 was reduced by RNAi. When CCAR1 mRNA and protein levels were specifically reduced in LNCaP cells infected with lentiviruses expressing a CCAR1 shRNA (shCCAR1), DHT-induced expression of well characterized AR target genes such as PSA (KLK3), TMPRSS2, KLK2, KLK4, SNAI2 (Slug), FKBP5, SLC16A6, and NKX3.1 was significantly inhibited compared with the results using a NS shRNA (Figure 2A and B). Similarly, a shRNA (shCCAR1 M1) targeting a different region of CCAR1 mRNA also efficiently inhibited DHT-induced expression of AR target genes (Supplementary Figure S4A and B). Similar results were also observed in CCAR1-depleted VCaP cells (Supplementary Figure S5A and B). In addition, DHT-induced expression of transiently transfected reporter genes derived by MMTV LTR, PSA and probasin gene regulatory regions in LNCaP cells was inhibited by the CCAR1-specific shRNA but not by shNS (Supplementary Figure S6A and B), suggesting that CCAR1 is required for the expression of AR target genes.

To confirm these findings and to assess the global effect of CCAR1 on the expression of androgen responsive genes, we performed genome-wide gene expression analysis in control (shNS) and CCAR1-depleted (shCCAR1) LNCaP cells (Figure 2C). Microarray analysis of LNCaP/shNS revealed that DHT upregulated expression of 342 genes and downregulated 495 genes (fold change ≥1.5, *P* < 0.05). After normalizing the data, we identified CCAR1-dependent genes that exhibited a fold change of greater than 1.4 between control shNS/DHT+ and shCCAR1/DHT+ LNCaP cells. Approximately 19.2% of the 837 differentially expressed genes were affected by CCAR1 depletion (Figure 2D), indicating that these were regulated by both DHT and CCAR1 and that the expression of a subset of DHT-regulated genes depends on CCAR1. Among the DHT upregulated genes, 86 genes (25.1%) including the AR target genes tested in qRT-PCR experiments (Figure 2B) were downregulated in LNCaP/shCCAR1 cells relative to control LNCaP/shNS cells (Figure 2E). Interestingly, among the DHT downregulated genes, 72 genes (14.5%) were derepressed in CCAR1-depleted cells (Figure 2F). For example, DHT-mediated repression of UGT2B15 and UGT2B17 genes, which are well-characterized AR-repressed genes (16), was derepressed on CCAR1 depletion (Figure 2G), suggesting an unexpected negative role of CCAR1 in DHT-regulated gene expression. In addition, we also found that 188 genes were upregulated or downregulated by CCAR1 depletion in the absence of DHT (Figure 2H). These genes represent candidate CCAR1 target genes that are independent of AR signaling. These significantly differentially expressed genes are depicted in heat maps (Supplementary Figures S7 and S8). Together, these results suggest that CCAR1 can positively and negatively regulate transcription of specific subsets of AR target genes.

### CCAR1 is required for tumorigenic potential of prostate cancer cells

**Figure 3.**
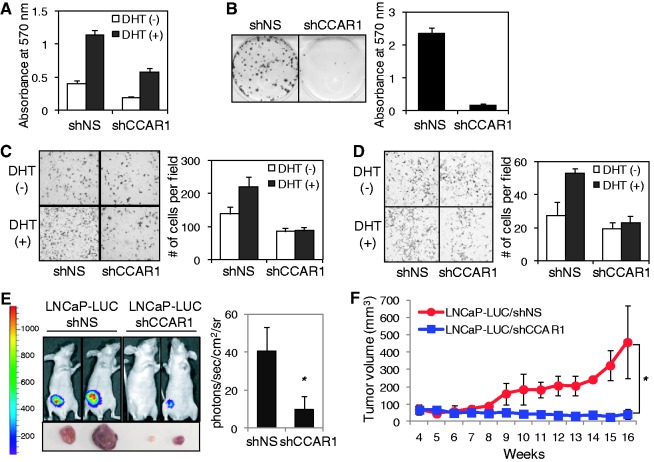
CCAR1 is required for the tumorigenic properties of prostate cancer cells. (**A**) Proliferation assay. LNCaP cells infected with lentiviruses expressing shNS or shCCAR1 were treated with 10 nM DHT or ethanol vehicle for 10 days. Cell viability was determined by MTT assay. Data are means ± SD (n = 6). (**B**, **C** and **D**) Colony formation, migration and invasion assays. LNCaP cells were infected with lentiviruses expressing shNS or shCCAR1. Representative images of anchorage-dependent colony formation, cell migration and invasion assays are shown on the left panel of each figure, and the quantitative analysis (n = 3, ±SD) is shown on the right panel of each figure. For colony formation assay, viable colonies were stained with crystal violet; after washing, the dye was extracted with 10% SDS solution and quantified by absorbance at 570 nm (B). Migration and invasion assays were performed using Transwell chambers without or with Matrigel as described under Supplementary Materials and Methods (C and D). (**E** and **F**) Effect of CCAR1 depletion on the growth of LNCaP xenograft tumors. LNCaP cells expressing shNS or shCCAR1 were injected subcutaneously in athymic male mice. Tumor growth was monitored for 16 weeks under treatment with DHT in drinking water. Representative bioluminescence images of tumor-bearing mice, and their tumors are shown on the left panel and the average signal intensity (n = 6, ±SD) of regions of interest (ROIs) is shown on the right panel (E). Tumor growth curves are shown (F). *P*-value was determined by Student’s *t*-test (*P < 0.05).

To investigate a potential role of CCAR1 in prostate tumorigenesis, we searched the public cancer microarray database ONCOMINE (http://www.oncomine.org) and found four suitable studies (Supplementary Figure S9). In these studies, expression of CCAR1 mRNA was higher in prostate carcinoma samples than in normal prostate gland samples, suggesting that CCAR1 might play an important role in the growth and tumorigenesis of prostate cancer cells. To investigate the possible role of CCAR1 in prostate cancer growth, we examined the effect of reduced CCAR1 levels on DHT-stimulated cell proliferation in LNCaP cells infected with lentiviruses expressing a shNS or CCAR1 shRNA (Figure 3A). DHT treatment stimulated proliferation of LNCaP/shNS cells, but DHT stimulation of LNCaP cell growth was attenuated by CCAR1 depletion. Similar results were observed in CCAR1-depleted VCaP cells (Supplementary Figure S5C). Furthermore, depletion of CCAR1 decreased the clonogenic survival of LNCaP cells (Figure 3B) and inhibited LNCaP cell migration and invasion (Figure 3C and D), suggesting that CCAR1 is required for cellular properties associated with the transformed phenotype of prostate cancer cells. To further investigate the role of CCAR1 in promoting prostate tumorigenesis, we assessed the effect of CCAR1 depletion on the growth of LNCaP xenograft tumors in nude mice injected with CCAR1-depleted (shCCAR1) cells or control (shNS) cells that had been engineered to stably express luciferase (LNCaP-LUC). The expression of luciferase in LNCaP-LUC cells was not affected by CCAR1 depletion (Supplementary Figure S10A and B). LNCaP-LUC/shNS tumors grew in DHT-treated mice; in contrast, DHT-stimulated tumor growth was almost abolished in mice injected with LNCaP-LUC/shCCAR1 cells (Figure 3E and F). Immunohistochemistry analysis using anti-CCAR1 antibody confirmed that CCAR1 expression was much weaker in tumor specimens derived from LNCaP-LUC/shCCAR1 grown in mice when compared with LNCaP-LUC/shNS tumors, indicating that CCAR1 depletion was maintained during the tumor formation in mice (Supplementary Figure 10C). Collectively, these results strongly suggest that CCAR1 plays a pivotal role in tumorigenic growth of prostate cancer cells.

### CCAR1 is required for AR transcription complex assembly on androgen-dependent enhancers and for chromatin looping between the PSA enhancer and promoter

**Figure 4.**
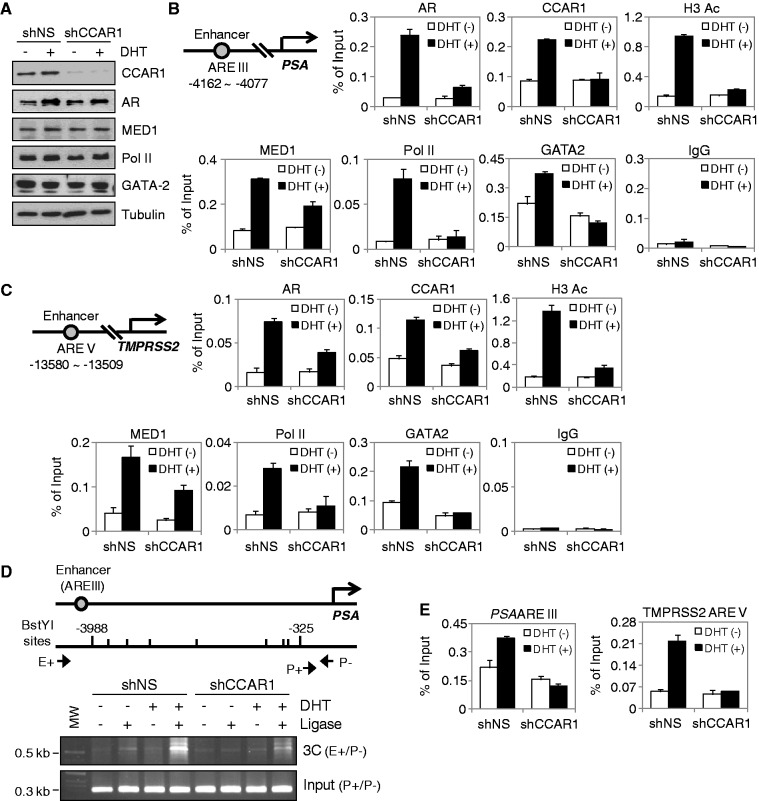
CCAR1 is required for optimal association of AR with target enhancers and AR-mediated chromatin looping and chromatin remodeling. (**A**, **B** and **C**) LNCaP cells expressing shNS or shCCAR1 were treated with or without 10 nM DHT for 16 h. Protein levels were monitored by immunoblot using the indicated antibodies (A). ChIP assays using the indicated antibodies were performed as described in Figure 1C. qPCR analyses were performed using primers specific for the PSA (B) and TMPRSS2 (C) enhancers. The results are shown as percentage of input and are means ± SD (n = 3). (**D**) 3C assays were performed using crosslinked, BstYI-digested chromatin from LNCaP cells expressing shNS or shCCAR1 treated with or without 10 nM DHT for 16 h. The ligated DNA was PCR amplified with primers as indicated. (**E**) FAIRE was conducted with LNCaP cells expressing shNS or shCCAR1 treated with or without 10 nM DHT for 16 h. qPCR analyses were performed using primers specific for the PSA and TMPRSS2 enhancers.

Recently, we have reported that CCAR1 plays an important role in transcription complex assembly on target promoters by providing a physical link between activated transcription factors and Mediator complexes and thereby facilitates recruitment of Pol II to the promoter of target genes (8). To further test the role of CCAR1 as an AR co-activator, we assessed the effect of reduced CCAR1 levels on transcription complex assembly on the PSA enhancer. Reduction of CCAR1 levels by shRNA had no measurable effect on the cellular levels of AR, MED1 and Pol II (Figure 4A). However, CCAR1 depletion severely affected the DHT-dependent recruitment of AR to the PSA enhancer, and the recruitment of MED1 and Pol II was greatly reduced (Figure 4B). Similar results were observed on the TMPRSS2 and KLK2 enhancers (Figure 4C and Supplementary Figure S11A). Interestingly, CCAR1 depletion also abolished RNA Pol II recruitment to the PSA promoter but caused only a modest reduction in AR occupancy on the promoter (Supplementary Figure S12). The hormone-induced level of histone H3 acetylation was also reduced by CCAR1 depletion (Figure 4B and C and Supplementary Figure S11). These results are consistent with the data that CCAR1 is required for AR-mediated transcription (Figure 2) and strongly suggest that CCAR1 is required for efficient binding of AR to its regulatory regions and thereby facilitates the occupancy of Mediator and Pol II on the regulatory regions of AR target genes.

Given previous findings that the pioneer factor GATA2, but not FoxA1, assists AR binding to the PSA and TMPRSS2 enhancers (5), and our findings that CCAR1 is required for optimal association of AR with the PSA, TMPRSS2 and KLK2 enhancers, we then investigated the effect of CCAR1 depletion on GATA2 recruitment to the enhancers. GATA2 recruitment to these enhancers was significantly decreased in CCAR1-depleted cells, both in the absence of ligand and, to a great extent, in the presence of androgen (Figure 4B and C). A similar recruitment pattern of GATA2 was observed on GATA2-dependent AR-binding sites of KLK2 gene (17) (Supplementary Figure S11A). CCAR1 depletion had no measurable effect on the level of GATA2 expression (Figure 4A). This suggests that CCAR1 plays necessary roles both in the basal recruitment of GATA2 to chromatin and in its recruitment following androgen stimulation.

In the presence of androgen, AR and its co-activators bind to the distal PSA enhancer, and the AR-co-activator complex communicates with the proximal PSA promoter through chromosomal looping (7). As CCAR1 is required for the recruitment of AR and Pol II to the PSA enhancer, we next asked whether CCAR1 can affect PSA enhancer/promoter loop formation. To this end, we performed 3C analysis, a technique used to detect physical long-range chromosomal interaction (12). The cross-linked chromatin from LNCaP cells treated with or without DHT for 16 h was digested with BstYI and ligated. After reversing crosslinking, PCR was performed with one primer in the PSA enhancer and another in the PSA promoter (primer E+/P−). Control PCR (primer P+/P−) for the level of input chromatin was equivalent under all conditions. As expected, the PSA enhancer and promoter interacted in a ligase-dependent manner, and this interaction was enhanced by DHT treatment (Figure 4D). However, depletion of CCAR1 severely reduced the interaction between the PSA enhancer and promoter, implicating its involvement in bridging between the enhancer and promoter of the PSA gene. Together, these results indicate that CCAR1 is required for chromatin looping and maximal occupancy of AR and Pol II at the PSA locus. To assess whether CCAR1 plays a direct role in mediating chromatin remodeling at AR-binding regions, we performed FAIRE analysis, a method for enriching nucleosome-depleted regions of the genome (13). CCAR1 depletion reduced androgen-inducible chromatin remodeling of AR-binding regions (Figure 4E), suggesting that CCAR1 is required for optimal euchromatic conditions at the enhancer of AR target genes.

### CCAR1 stabilizes the association between AR and GATA2

**Figure 5.**
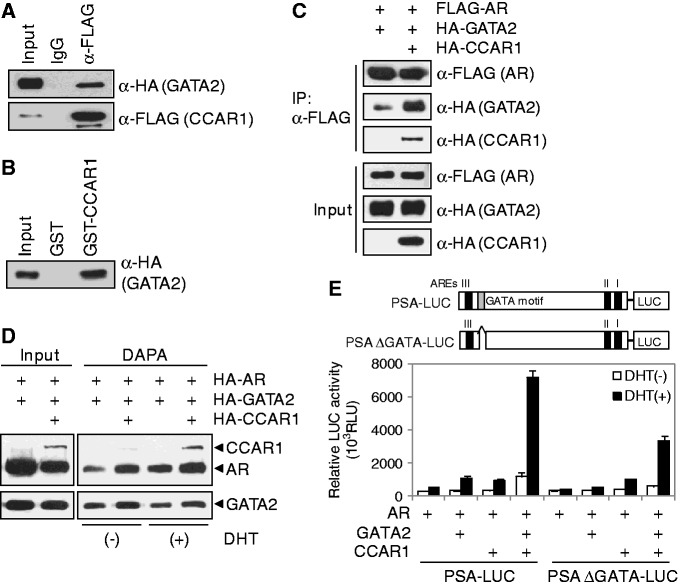
CCAR1 stabilizes the association between AR and GATA2 and enhances their DNA binding. (**A**) 293T cell extracts transfected with pSG5.HA-GATA2 and pSG5.FLAG-CCAR1 were immunoprecipitated with anti-FLAG antibody or normal IgG. Immunoprecipitated CCAR1 and coimmunoprecipitated GATA2 were detected by the indicated antibodies. (**B**) *In vitro* translated HA-tagged GATA2 was incubated with recombinant GST-CCAR1, and bound proteins were analyzed by immunoblot with anti-HA antibody. (**C**) 293T cells were transfected with expression vectors as indicated. Cell lysates were immunoprecipitated with anti-FLAG antibody. Immunoprecipitated AR and coimmunoprecipitated GATA2 and CCAR1 were detected by the indicated antibodies. (**D**) DAPA was performed with biotinylated PSA enhancer fragments and 293T cell extracts transfected with the indicated expression vectors in the presence or absence of DHT. Proteins captured by biotinylated probes were analyzed by immunoblot with anti-HA antibody. (**E**) Transient transfections using PSA-LUC reporters and expression vectors for AR, GATA2 and CCAR1 were performed as described in Figure 1B.

The aforementioned observations prompted us to examine the interaction and cooperation between CCAR1 and GATA2. In CoIP experiments, GATA2 was efficiently coimmunoprecipitated with CCAR1 (Figure 5A). In addition, *in vitro* GST pull-down assays confirmed the interaction between CCAR1 and GATA2 (Figure 5B), suggesting that CCAR1 interacts directly with GATA2. Since CCAR1 interacts with both AR and GATA2, we next mapped the CCAR1 domains involved in the interaction with AR and GATA2 in GST pull-down assays (Supplementary Figure S13). AR interacted with the N-terminal domain of CCAR1 (aa 1–249); in contrast, GATA2 bound to the central domain of CCAR1 (aa 203–660). Thus, AR and GATA2 interact with different domains of CCAR1, suggesting that CCAR1 might interact simultaneously with AR and GATA2. Furthermore, CCAR1 significantly increased the amount of GATA2 that coimmunoprecipitated with AR in CoIP experiments (Figure 5C). To test whether CCAR1 can stabilize the interaction between AR and GATA2 on DNA, we next performed DAPA assays. A biotinylated PSA enhancer probe was incubated with 293T cell extracts transiently transfected with AR, GATA2 and CCAR1 expression vectors, and then the DNA–protein complexes were precipitated by streptavidin beads. As shown in Figure 5D, AR binding to the enhancer was increased in the presence of DHT. Furthermore, AR and GATA2 binding to DNA was increased by enforced expression of CCAR1 both in the presence and absence of DHT, suggesting that CCAR1 increases DNA-binding activities of AR and GATA2 by stabilizing the interaction between AR and GATA2 on the PSA enhancer. In reporter gene assays using PSA-LUC constructs, CCAR1 and GATA2 synergistically enhanced the transcriptional activity of AR (Figure 5E). In contrast, deletion of the GATA motif within the PSA enhancer region dramatically decreased GATA2 effect on AR activity and the synergy between GATA2 and CCAR1, demonstrating the functional importance of the interaction among AR, GATA2, and CCAR1 on the PSA enhancer. Taken together, our results suggest that CCAR1 may function as adaptor protein that stabilizes the interaction of GATA2 and AR and possibly maintains the integrity of AR transcription complex.

## DISCUSSION

### Role of CCAR1 in AR-mediated transcription in prostate cancer cells

AR-mediated androgen signaling plays an important role in onset and progression of prostate cancer (1–3). In this study, we show CCAR1 as a key factor in androgen signaling in prostate cancer cells. CCAR1 interacted with AR and enhanced the transcriptional activity of AR (Figure 1 and Supplementary Figure S1). CCAR1 is recruited to the enhancers of endogenous AR target genes (Figure 1 and Supplementary Figures S3 and S11); and depletion of CCAR1 caused reduction in DHT-induced expression of a subset of endogenous AR target genes (Figure 2 and Supplementary Figures S4 and S5), significantly reduced hormone-dependent recruitment of AR, MED1 and Pol II to the enhancers (Figure 4 and Supplementary Figure S11) and attenuated hormone-dependent growth of prostate cancer cells (Figure 3A and Supplementary Figure S5C). Furthermore, CCAR1 silencing reduced the migration, invasion, survival and tumorigenic potential of prostate cancer cells (Figure 3). These results firmly established CCAR1 as an AR co-activator that has an important physiological role in prostate cancer cell growth and tumorigenesis.

A large number of proteins have been reported as AR co-regulators (4), but whether specific co-regulators are involved in mediating AR control of specific target genes has not been clearly addressed. By global gene expression analysis, we found that CCAR1 regulates positively or negatively the transcriptional efficiency of specific subsets of AR target genes (Figure 2 and Supplementary Figures S7 and S8). Depletion of CCAR1 affected the expression of 161 (19.2%) of 837 DHT-regulated genes. Specifically, CCAR1 functioned as a co-activator for 86 of 87 genes that were induced by DHT and affected by CCAR1 depletion; and CCAR1 served as a co-repressor for 72 of 74 genes that were repressed by DHT and affected by CCAR1 depletion. Thus, in most cases, CCAR1 facilitated, rather than opposed, the positive or negative regulation of genes by DHT, suggesting that CCAR1 functioned as a co-activator for androgen-activated genes and as a co-repressor in support of androgen-induced gene repression.

### Molecular mechanism of CCAR1 co-regulator function

**Figure 6.**
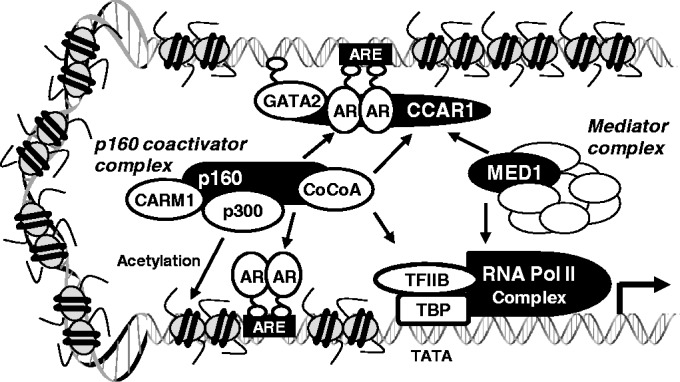
The proposed role of CCAR1 in chromatin loading and assembly of AR transcription complex. CCAR1 is recruited to GATA2-dependent AR-binding sites at least in part through interaction with DNA-bound GATA2 and AR and stabilizes their association with DNA, thereby increasing their occupancy on AR-binding sites. The recruited CCAR1 possibly provides a physical link between DNA-bound AR and Mediator complex and facilitates recruitment of p160 co-activator complex, and thereby facilitating transcription complex assembly, chromatin looping and chromatin remodeling. For example, CCAR1 facilitates AR and GATA2 recruitment and stabilizes their occupancies on the distal PSA enhancer (ARE III) containing a GATA2-dependent ARE. CCAR1 also facilitates recruitment of Mediator complex and Pol II to AR-bound PSA enhancer (ARE III) and promoter (ARE I and ARE II). CCAR1 provides a physical link in AR transcription complex, thereby leading to chromatin looping and enhancing communication between the PSA enhancer and promoter.

Genome-wide ChIP-on-chip and ChIP-Seq studies identified a highly significant overlap of AR and GATA2-binding sites in androgen target genes and showed that GATA2 acts as a pioneer factor in the recruitment of AR to AR binding sites (5,6,17). In this study, we report a novel mechanism for AR-mediated transcription involving the interplay between AR, GATA2 and CCAR1. Our ChIP analyses showed that recruitment of AR and GATA2 to the PSA, TMPRSS2 and KLK2 enhancers was decreased by CCAR1 depletion (Figure 4B and C and Supplementary Figure S11A). In line with these observations, MED1 and Pol II failed to bind to the enhancers, and consequently, the expression of PSA, TMPRSS2 and KLK2 was significantly reduced (Figure 2B and Supplementary Figure S4). These results suggest that AR and GATA2 bind to the enhancer more efficiently when complexed with CCAR1. Indeed, CCAR1 stabilized the interaction between AR and GATA2 by associating with both proteins (Figure 5C) and enhanced their DNA-binding activity (Figure 5D). Although GATA2 is required for PSA, TMPRSS2 and KLK2 expression (5,18), GATA2 does not play a significant role in androgen-regulated FKBP5 expression (19). Interestingly, CCAR1 depletion did not affect AR binding but greatly reduced MED1 and Pol II loading on the FKBP5 enhancer (Supplementary Figure S11B), which is similar to our previous finding that inhibition of CCAR1 recruitment to the pS2 promoter had no observable effect on recruitment of ERα but compromised the hormone-dependent recruitment of Mediator complex and Pol II to the promoter (8). Similarly, CCAR1 depletion caused only a modest reduction in AR recruitment to the PSA promoter containing GATA2-independent AREs (Supplementary Figure S12). Thus, we propose that CCAR1 has a dual function in regulating AR signaling: first as an adaptor protein by stabilizing the association between AR and GATA2 and increasing their occupancy on AR-binding sites, and second as a co-activator by facilitating recruitment of Mediator complex and Pol II to AR-binding sites by providing a physical link between DNA-bound AR and the Mediator complex, and thereby facilitating transcription complex assembly, chromatin looping and chromatin accessibility (Figure 6).

The exact mechanism of CCAR1-mediated gene repression is unclear. However, it is becoming apparent that many co-activators and co-repressors can carry out either function in a gene-specific manner. For example, GRIP1, a member of p160 co-activator family, has been shown to regulate positively or negatively the transcriptional efficiency of specific subsets of NR target genes. GRIP1 interacts directly with hormone-activated NRs, recruits secondary co-activators (p300, CARM1 and CoCoA) (Figure 6), and acts synergistically with secondary co-activators to enhance NR function (11). However, GRIP1 can also serve as a platform for recruitment of downstream co-repressors such as RIP140 and Suv4-20h1.1 to specific subsets of NR target genes (20,21). G9a, a histone H3K9 methyltransferase, not only acts as a co-repressor but also has been shown to function as a co-activator for NRs. As a co-activator, G9a acts as a molecular scaffold to facilitate recruitment of co-activators CARM1 and p300 to GR target genes, and G9a co-activator function, in contrast to its co-repressor function, is independent of its catalytic H3K9 methyltransferase activity (22,23). DBC1, a NR co-activator (9,10), was also found to be involved in transcriptional repression (24). DBC1 associates with COUP transcription factor 1 (COUP-TFI) complex, stabilizes the interaction between NCoR and COUP-TFI, and thereby contributes to COUP-TFI-mediated transcriptional repression. Thus, the positive or negative actions of co-regulators are likely determined by the gene-specific regulatory environment, chromatin architecture and combinatorial interactions between different co-regulators. A recent proteomics study found a few proteolytic fragments of CCAR1 in immunopurified SIRT7 complexes (25), suggesting that CCAR1 might be associated with some forms of the SIRT7 complex. SIRT7, a NAD-dependent protein deacetylase, deacetylates histone H3K18 and promotes transcriptional repression (26). Thus, it will be interesting to test whether SIRT7 is involved in CCAR1-mediated repression.

### CCAR1 as a potential therapeutic target for prostate cancer

AR plays a critical role in all phases of prostate cancer, including onset, androgen-dependent tumor growth and transition to androgen-independence of prostate cancer (1,2). Interestingly, several recent studies have shown that the program of gene expression regulated by AR in androgen-independent prostate cancer is distinct from the androgen-regulated program in androgen-dependent prostate cancer (27,28). In addition, the AR transcription program in primary human tissue is divergent from that in cultured prostate cancer cells (27). These differences in AR transcriptional program between androgen-dependent prostate cancer and androgen-independent prostate cancer or between cultured cells and primary human tissue could be explained by genetic or epigenetic alterations in co-regulators and pioneer factors to modulate AR activity. For example, recent reports demonstrated that high expression of GATA2 and AIB1/SRC3 is predictive of poor outcome in prostate cancer and modulates the expression of key androgen-regulated genes that have potential roles in the transition of prostate cancer cells to aggressive phenotype (18,29). Co-regulators play crucial roles in modulating AR activity and consequently may be important in regulating aberrant activity of AR during prostate cancer progression (1,4). In this study, we showed that CCAR1 is required for growth and tumorigenic properties of prostate cancer cells. Importantly, we further demonstrated that CCAR1 contributes to chromatin looping and AR transcription complex assembly by stabilizing AR-GATA2 association on chromatin and facilitating MED1 and Pol II recruitment to AR-binding sites. A recent study showed that MED1 plays an essential role in chromatin looping at the castration-resistant prostate cancer-specific AR target UBE2C gene locus (30). Given that CCAR1 is required for optimal recruitment of MED1 to AR-binding sites, it will be interesting to test whether CCAR1 also contributes to UBE2C locus looping and castration-resistant prostate cancer progression. Understanding the co-activators involved in the mechanisms of AR–DNA interaction and AR transcription complex assembly will provide useful information for developing therapeutic drugs for the treatment of prostate cancer. Our study thus suggests CCAR1 as a potential therapeutic target for prostate cancer.

## SUPPLEMENTARY DATA

Supplementary Data are available at NAR Online, including [31–37].

## FUNDING

Samsung Biomedical Research Institute (SBRI) grant [GE1-B2-061]; Korean Foundation for Cancer Research [CB-2011-04-01]; Basic Science Research Program through the National Research Foundation of Korea funded by the Ministry of Education, Science and Technology [2010-0021428]; and Korea Healthcare Technology R&D Project funded by the Ministry for Health & Welfare [A092255]; Postdoctoral training grant for translational research from Samsung Medical Center [SMX1131141 to W.-Y.S.]. Funding for open access charge: SBRI grant [GE1-B2-061].

*Conflict of interest statement*. None declared.

## Supplementary Material

Supplementary Data
