# Supplementary material for: CCAR1 promotes chromatin loading of androgen receptor (AR) transcription complex by stabilizing the association between AR and GATA2
Source: Nucleic Acids Res. 2013 Jul 25;41(18):8526–36. doi: 10.1093/nar/gkt644 (PMC3794601; doi:10.1093/nar/gkt644)
Supplement: Supplementary Data [file supp_41_18_8526__index.html]

CCAR1 promotes chromatin loading of androgen receptor (AR) transcription complex by stabilizing the association between AR and GATA2 — CCAR1 promotes chromatin loading of androgen receptor (AR) transcription complex by stabilizing the association between AR and GATA2 — Supplementary Data 

# CCAR1 promotes chromatin loading of androgen receptor (AR) transcription complex by stabilizing the association between AR and GATA2

## 

files

**Files in this Data Supplement:**

- Supplementary Data - pdf file
