# Supplementary material for: CCAR1 promotes chromatin loading of androgen receptor (AR) transcription complex by stabilizing the association between AR and GATA2
Source: Nucleic Acids Res. 2013 Jul 25;41(18):8526–36. doi: 10.1093/nar/gkt644 (PMC3794601; doi:10.1093/nar/gkt644)

Supplementary Data

Supplementary Materials and Methods

Plasmids and antibodies

cDNA encoding AR from pSG5-AR was cloned into pSG5.HA, pSG5.FLAG, and pGEX-4T-1, and cDNA encoding GATA2 (Open Biosystems) was cloned into pSG5.HA and pGEX-4T-1. To generate a lentiviral vector expressing luciferase, cDNA encoding LUC2P from pGL4.35 (Promega) was cloned into pHR.CMV.FLAG.IRES-Hygro (1). cDNAs encoding CCAR1 fragments and AR fragments were cloned into pSG5.HA. The following plasmids were described previously: pSG5.HA-CCAR1, pSG5.FLAG-CCAR1, pGEX-5X-1-CCAR1, pSG5.HA-DBC1, MMTV-LUC, pHR.CMV.puro.Sin8-shNS, and pHR.CMV.puro.Sin8-shCCAR1 (1,2). The lentiviral pLKO.1-puro vector (pLKO.1-shCCAR1 M1) containing the shRNA against CCAR1 (TRCN0000056005) was purchased from Sigma-Aldrich. Lentiviral particles were generated as described previously (2). PSA-LUC (pGL3_PSA540) and Probasin-LUC (ARR3-tk-luc/tk81-PB3) reporters were kind gifts from Gerhard Coetzee (University of Southern California). PSAΔGATA-LUC (lacking GATA binding motif) was generated with the QuikChange site-directed mutagenesis kit (Stratagene). The following antibodies were used in this study: anti-AR antibody N-20, anti-CCAR1 antibody C-20, anti-DBC1 antibody H2, anti-MED1 antibody C-19, anti-RNA polymerase II antibody 8WG16, anti-GATA2 antibody H-116, and anti-tubulin antibody TU-02 (Santa Cruz Biotechnology); anti-FLAG monoclonal antibody (M2) and anti-FLAG M2 agarose (Sigma); anti-HA antibody 3F10 (Roche); anti-CCAR1 antibody 435A and anti-DBC1 antibody 434A (Bethyl Laboratories); anti-acetyl histone H3 antibody 06-599 (Millipore); anti-PSA antibody A0562 (Dako); anti-TMPRSS2 antibody 3209-1 (Epitomics); anti-luciferase antibody C-12 (Santa Cruz Biotechnology).

Protein interaction assays and immunoblot

For GST pull-down assays, HA epitope-tagged proteins were synthesized in vitro by using TNT-Quick coupled transcription/translation system (Promega) and incubated with immobilized GST-fusion proteins. After washing, bound proteins were analyzed by immunoblot with anti-HA antibody. For coimmunoprecipitation (CoIP) assays, 293T or LNCaP cell extracts were immunoprecipitated by specific antibodies or control IgG and protein G Dynabeads (Invitrogen) as indicated in figure legends.

Gene expression analysis by Affymetrix microarray

LNCaP cells expressing shNS or shCCAR1 were treated with ethanol or 10 nM DHT for 24 hr, and total RNA was isolated using the RNeasy mini kit (Qiagen). The integrity of RNA was analyzed using an Agilent 2100 Bioanalyzer. Two independent biological replicates were assayed for each sample. The microarrays were performed following the Affymetrix standard protocol. Briefly, total RNA was reverse-transcribed, and the double stranded cDNAs were used to generate biotin-labeled cRNA. These synthetic cRNAs were hybridized onto the GeneChip Human Gene 1.0 ST array (Affymetrix). The array was then scanned using Affymetrix GeneChip Scanner 3000 7G, and image analysis was performed by Affymetrix GCOS Software. The array data was summarized and normalized with Robust Multi-array analysis method by the software Expression Console (Affymetrix). Using normalized log2 intensities, we identified androgen-regulated genes by comparing the differential expression between LNCaP/shNS (DHT-) and LNCaP/shNS (DHT+) samples. The cutoffs for

differentially expressed genes were $p<0.05$ and fold change of 1.5 or greater for the entire datasets. Among the androgen-regulated genes, we identified CCAR1-dependent genes that exhibited a fold change of greater than 1.4 and a p value of less than 0.05. The data have been deposited in the Gene Expression Omnibus (GEO) database, www.ncbi.nlm.nih.gov/geo (accession no. GSE45033).

MTT, colony formation, migration, invasion assays

LNCaP cells were infected with lentivirus expressing a NS or CCAR1 shRNA. LNCaP cells were plated at a density of $2x10^4$ cells/well in 24-well plates and grown in the presence or absence of 10 nM DHT. Cell proliferation was determined by MTT assays (Promega). For colony formation assay, LNCaP cells were plated at a density of $2x10^3$ cells/well in 6-well plates and grown until colonies appeared. Cells were washed with PBS, fixed with methanol, and stained with 0.1% crystal violet for pictures. The stained cells were solubilized in 10% SDS, and absorbance was measured at 570 nm. Cell migration and invasion were determined by Transwell (Costar) migration and invasion assays. LNCaP cells were precultured in serum-free medium for 48 h. For migration assay, $5x10^4$ cells were seeded in serum-free medium in the upper chamber, and the lower chamber was filled with RPMI1640 containing 5% FBS. After 48 h, the non-migrating cells on the upper chambers were carefully removed with a cotton swab, and migrated cells underside of the filter stained and counted in nine different fields. Matrigel invasion assays were performed as described previously (3) using Transwell inserts (Costar) coated with Matrigel (BD Biosciences)/fibronectin (Sigma).

Xenograft experiments

LNCaP-LUC cells ($3x10^6$) infected with lentivirus expressing a NS or CCAR1 shRNA were suspended in 100 μl Matrigel/PBS (50:50 mixture) and injected subcutaneously into the right flank of 6-week-old male athymic BALB/c nu/nu mice (Orient Bio, Korea). Each experimental group contained ten mice. The drinking water was supplemented with DHT at a concentration of 1 μg/ml to stimulate tumor growth. Tumors were measured every week using a digital caliper and the volumes were calculated according the formula: Volume = $\pi/6 \times$ the largest diameter $\times$ (the smallest diameter)^2. For in vivo bioluminescence imaging, mice were anesthetized and given 150 μg/g of D-luciferin (Xenogen) in PBS by intraperitoneal injection. Fifteen minutes after injection, bioluminescence was imaged with the IVIS Spectrum Imaging System (Xenogen). The bioluminescence intensity was expressed as photon flux (photons/sec/cm^2/Steradian). All animal experiments were conducted with the approval of the Institutional Animal Care and Use Committee of Laboratory Animal Research Center at Samsung Biomedical Research Institute.

Immunohistochemical staining

Immunohistochemical staining was performed according to previously described protocol (3). Tumors from LNCaP-LUC xenografts were excised from the mice and fixed in buffered paraformaldehyde solution. The tumors were dehydrated and embedded in paraffin. Tissue sections (5 μm) mounted on slides were deparaffinized with xylene, rehydrated in serial dilutions of alcohol, and immersed in peroxidase-blocking solution (Dako) to quench endogenous peroxidase activity. For antigen retrieval, sections were microwaved in Target Retrieval Solution (Dako) for 15 min. Sections were incubated with anti-CCAR1 antibodies for 3 hr, washed with TBS-T, and then incubated with a horseradish peroxidase-labelled secondary anti-rabbit antibody (EnVision Detection System, Dako) for 30 min. DAB was used as a chromogen. All sections were counterstained with haematoxylin and eosin, rehydrated and mounted. Digital images of tissue sections were captured using the Aperio

ScanScope XT slide scanner (Aperio Technologies).

DNA affinity precipitation (DAPA) assays

The PSA enhancer region (nucleotides -4198~-3795 relative to transcription start site) containing ARE III and GATA binding motif was amplified by PCR with biotinylated primers. 293T cells were transfected with AR, GATA2, and CCAR1 expression vectors and lysed by sonication in DAPA buffer (20 mM HEPES, pH 7.9, 50 mM KCl, 1.5 mM $MgCl_2$, 0.2 mM $ZnCl_2$, 1 mM DTT, 10% glycerol, and 0.2% of NP-40). Cell extracts were incubated with 5 μg of biotinylated PSA enhancer DNA fragments and 5 μg of poly(dI-dC) for 4 hr in the presence or absence of DHT (0.5 μM). DNA-bound proteins were collected with streptavidin-agarose beads (Pierce) for 1 hr, washed with DAPA buffer, separated with SDS-PAGE, and identified by immunoblot.

Statistical analysis

Statistical significance was estimated by unpaired, two-tailed Student's t-test. *p* values are stated in the figure legends.

Primer sets used in this study

ChIP qPCR was performed using following forward (F) and reverse (R) primers (nucleotides relative to transcription start site): PSA enhancer (ARE III, -4162~-4077), 5'-TGG GAC AAC TTG CAA ACC TG-3' (F) and 5'-CCA GAG TAG GTC TGT TTT CAA TCC A-3' (R) (4); PSA promoter (ARE I & II, -230~-143), 5'-CCT AGA TGA AGT CTC CAT GAG CTA CA-3' (F) and 5'-GGG AGG GAG AGC TAG CAC TTG-3' (R) (4); TMPRSS2 enhancer (ARE V, -13580~-13509), 5'-CCT AGA TGA AGT CTC CAT GAG CTA CA-3' (F) and 5'-GAC ATA CGC CCC ACA ACA GA-3' (R) (5); KLK2 enhancer (ARE II, -3840~-3722), 5'-GGT TGA AAG CAG ACC TAC TCT GG-3' (F) and 5'-AGA TCT AGG TTT GCT TAC TGC CTT AG-3' (R) (6); FKBP5 enhancer (ARE VIII & IX, +126322~+126509), 5'-GGA GCC TCT TTC TCA GTT TTG-3' (F) and 5'-CAA TCG GAG TGT AAC CAC ATC-3' (R) (7). 3C qPCR was performed using following primers (nucleotide position relative to transcription start site): PSA 3C E+ (-4292), 5'-ATG TTC ACA TTA GTA CAC CTT GCC-3' (4); PSA 3C P- (-19), 5'-GCC CTA TAA AAC CTT CAT TCC CC-3' (4); PSA Input P+ (-317), 5'-GCA CGT GAG GCT TTG TAT GAA GAA-3'. qRT-PCR was performed using following forward (F) and reverse (R) primers: β-actin (2); PSA (KLK3), 5'-TCA CAG CTG CCC ACT GCA TCA-3' (F) and 5'-AGG TCG TGG CTG GAG TCA TC-3' (R) (8); TMPRSS2, 5'- CCT GCA GGG ACA TGG GCT ATA-3' (F) and 5'- CCG GCA CTT GTG TTC AGT TTC-3' (9); KLK2, 5'-GCT GCC CAT TGC CTA AAG AAG-3' (F) and 5'-TGG GAA GCT GTG GCT GAC A-3' (R) (9); FKBP5, 5'- AGG CTG CAA GAC TGC AGA TC-3' (F) and 5'- CTT GCC CAT TGC TTT ATT GG-3' (R) (9); KLK4, 5'-GGC ACT GGT CAT GGA AAA CGA-3' (F) and 5'-TCA AGA CTG TGC AGG CCC AGC C-3' (R); SNAI2 (Slug), 5'-GCC TCC AAA AAG CCA AAC TA-3' (F) and 5'-CAC AGT GAT GGG GCT GTA TG-3' (R); SLC16A6, 5'- ACA TCT TCA TTC AGA GCA TAG C-3' (F) and 5'- GTC CCA TCT TAC ACG GTC TC-3' (R); NKX3.1, 5'- CCG AGC CAG AAA GGC ACT TGG G-3' (F) and 5'- AGC GCT TCT GCG GCT GCT TA-3' (R); UGT2B15, 5'- GTG TTG GGA ATA TTA TGA CTA CAG TAA C-3' (F) and 5'- GGG TAT GTT AAA TAG TTC AGC CAG T-3' (R); UGT2B17, 5'-TGA CTT TTG GTT TCA AGC ATA-3' (F) and 5'-TTC CAT TTC CTT AGG CAA GGG-3' (R)

Supplementary References

1. Yu, E.J., Kim, S.H., Heo, K., Ou, C.Y., Stallcup, M.R. and Kim, J.H. (2011) Reciprocal roles of DBC1 and SIRT1 in regulating estrogen receptor α activity and co-activator synergy. *Nucleic Acids Res*, **39**, 6932-6943.
2. Kim, J.H., Yang, C.K., Heo, K., Roeder, R.G., An, W. and Stallcup, M.R. (2008) CCAR1, a key regulator of mediator complex recruitment to nuclear receptor transcription complexes. *Mol Cell*, **31**, 510-519.
3. Kim, S.H., Kim, J.H., Yu, E.J., Lee, K.W. and Park, C.K. (2012) The overexpression of DBC1 in esophageal squamous cell carcinoma correlates with poor prognosis. *Histol Histopathol*, **27**, 49-58.
4. Wang, Q., Carroll, J.S. and Brown, M. (2005) Spatial and temporal recruitment of androgen receptor and its coactivators involves chromosomal looping and polymerase tracking. *Mol Cell*, **19**, 631-642.
5. Wang, Q., Li, W., Liu, X.S., Carroll, J.S., Janne, O.A., Keeton, E.K., Chinnaiyan, A.M., Pienta, K.J. and Brown, M. (2007) A hierarchical network of transcription factors governs androgen receptor-dependent prostate cancer growth. *Mol Cell*, **27**, 380-392.
6. Andreu-Vieyra, C., Lai, J., Berman, B.P., Frenkel, B., Jia, L., Jones, P.A. and Coetzee, G.A. (2011) Dynamic nucleosome-depleted regions at androgen receptor enhancers in the absence of ligand in prostate cancer cells. *Mol Cell Biol*, **31**, 4648-4662.
7. Makkonen, H., Kauhanen, M., Paakinaho, V., Jaaskelainen, T. and Palvimo, J.J. (2009) Long-range activation of FKBP51 transcription by the androgen receptor via distal intronic enhancers. *Nucleic Acids Res*, **37**, 4135-4148.
8. Lee, D.Y., Northrop, J.P., Kuo, M.H. and Stallcup, M.R. (2006) Histone H3 lysine 9 methyltransferase G9a is a transcriptional coactivator for nuclear receptors. *J Biol Chem*, **281**, 8476-8485.
9. Ianculescu, I., Wu, D.Y., Siegmund, K.D. and Stallcup, M.R. (2012) Selective roles for cAMP response element-binding protein binding protein and p300 protein as coregulators for androgen-regulated gene expression in advanced prostate cancer cells. *J Biol Chem*, **287**, 4000-4013.
10. Arredouani, M.S., Lu, B., Bhasin, M., Eljanne, M., Yue, W., Mosquera, J.M., Bubley, G.J., Li, V., Rubin, M.A., Libermann, T.A. *et al.* (2009) Identification of the transcription factor single-minded homologue 2 as a potential biomarker and immunotherapy target in prostate cancer. *Clin Cancer Res*, **15**, 5794-5802.
11. Grasso, C.S., Wu, Y.M., Robinson, D.R., Cao, X., Dhanasekaran, S.M., Khan, A.P., Quist, M.J., Jing, X., Lonigro, R.J., Brenner, J.C. *et al.* (2012) The mutational landscape of lethal castration-resistant prostate cancer. *Nature*, **487**, 239-243.
12. Vanaja, D.K., Cheville, J.C., Iturria, S.J. and Young, C.Y. (2003) Transcriptional silencing of zinc finger protein 185 identified by expression profiling is associated with prostate cancer progression. *Cancer Res*, **63**, 3877-3882.
13. Luo, J.H., Yu, Y.P., Cieply, K., Lin, F., Deflavia, P., Dhir, R., Finkelstein, S., Michalopoulos, G. and Becich, M. (2002) Gene expression analysis of prostate cancers. *Molecular carcinogenesis*, **33**, 25-35.

Supplementary Figure Legends

Figure S1. CCAR1 interacts with AR. (A) 293T cells transfected with pSG5.HA-CCAR1 and pSG5.FLAG-AR were treated with ethanol or 10 nM DHT. Cell lysates were immunoprecipitated with anti-FLAG antibody or normal IgG. Immunoprecipitated AR and coimmunoprecipitated CCAR1 were detected by the indicated antibodies. (B) In vitro translated HA-tagged AR was incubated with recombinant GST-CCAR1 in the presence or

absence of 0.3 μM DHT. Bound proteins were analyzed by immunoblot with anti-HA antibody. (C) Schematic representation of full-length AR and deletion mutants tested in GST pull-down assays. AF, activation function; DBD, DNA binding domain; LBD, ligand binding domain. (D and E) CCAR1 interacts with the C-terminal domain of AR. GST pull-down assays were performed as describe above using in vitro translated AR fragments.

Figure S2. Synergy between CCAR1 and DBC1. Transient transfections were performed as described in Figure 1B. Expression vectors: pSG5.HA-DBC1 (400 ng), pSG5.HA-CCAR1 (200 and 400 ng). Data are means ± SD (n=3).

Figure S3. Recruitment of CCAR1 to AR target genes in VCaP cells. Cross-linked, sheared chromatin from VCaP cells treated with or without 10 nM DHT (16 hr) was immunoprecipitated with the indicated antibodies. qPCR analyses were performed using primers specific for the PSA (A) and TMPRSS2 (B) enhancers. The results are shown as percentage of input and are means ± SD (n=3).

Figure S4. CCAR1 is required for androgen-induced expression of AR target genes. (A) LNCaP cells infected with lentiviruses encoding a non-specific (shNS) or CCAR1 shRNA (shCCAR1 M1) were treated with 10 nM DHT or ethanol vehicle for 24 hr. Total RNA was examined by real-time qRT-PCR analysis with primers specific for the indicated mRNAs. Results shown were normalized to β-actin mRNA levels and are means ± SD (n=3). (B) Protein levels were monitored by immunoblot using the indicated antibodies.

Figure S5. CCAR1 is required for DHT-induced gene expression and hormone-dependent growth of TMPRSS2-ERG gene fusion-positive VCaP cells. (A and B) VCaP cells infected with lentiviruses encoding a non-specific (shNS) or CCAR1 shRNA (shCCAR1) were treated with 10 nM DHT or ethanol vehicle for 24 hr. Total RNA was examined by real-time qRT-PCR analysis with primers specific for the indicated mRNAs (A). Results shown were normalized to β-actin mRNA levels and are means ± SD (n=3). Protein levels were monitored by immunoblot using the indicated antibodies (B). (C) Proliferation assay. VCaP cells infected with lentiviruses expressing shNS or shCCAR1 were treated with 10 nM DHT or ethanol vehicle for 14 days. Cell viability was determined by MTT assay. Data are means ± SD (n=6).

Figure S6. CCAR1 is required for DHT-induced expression of AR-regulated reporter gene. (A) LNCaP cells infected with lentiviruses encoding a NS or CCAR1 shRNA were transfected with indicated reporters, treated or untreated with DHT for 48 hr, and harvested for luciferase assays. Results shown are mean and SD of triplicate points. (B) Protein levels were monitored by immunoblot using the indicated antibodies.

Figure S7. Heat map diagram of CCAR1-dependent genes that are regulated by androgen. (A) Heat map of CCAR1-dependent genes that are induced by DHT. (B) Heat map of CCAR1-dependent genes that are repressed by DHT.

Figure S8. Heat map diagram of CCAR1-dependent genes. (A) Heat map of downregulated genes by CCAR1 shRNA. (B) Heat map of upregulated genes by CCAR1 shRNA.

Figure S9. Bioinformatics analysis of CCAR1 mRNA expression in human prostate carcinoma samples using database at ONCOMINE (http://www.oncomine.org). *p* value was

determined by Student's t-test. Arredouani Prostate study monitored gene expression profiles of 14 treatment-naive, intermediate-grade (Gleason score 6-7), and localized prostate cancer tissues (10); Grasso Prostate study performed gene expression microarray analysis of 59 treatment-naive, high grade, localized prostate cancer tissues and 35 lethal, heavily pre-treated metastatic castration-resistant prostate cancer tissues (11); Vanaja Prostate study monitored gene expression profiles of 11 treatment-naive, intermediate grade (Gleason score 6) prostate cancer tissues, 12 treatment-naive, high grade (Gleason score 9) prostate cancer tissues, and 5 metastatic prostate cancer tissues (12); Luo Prostate study performed a comprehensive gene expression analysis on 30 prostate tissues of various levels of invasiveness (ranging from localized prostate cancer to metastatic prostate cancer) and Gleason grades (combined scores 4-9) (13).

Figure S10. Depletion of CCAR1 by shRNA in LNCaP-LUC cells and LNCaP-LUC xenograft tumors. (A and B) CCAR1 depletion does not affect the expression and activity of luciferase driven by the CMV promoter in LNCaP-LUC cells. Protein levels in LNCaP-LUC cells infected with lentiviruses encoding a non-specific (shNS) or CCAR1 shRNA (shCCAR1) were monitored by immunoblot using the indicated antibodies (A). Luciferase activity was also measured (B). Results shown are mean and SD of triplicate points. (C) Immunohistochemical analysis of CCAR1 expression in tumor specimens derived from the LNCaP xenograft model. At the end of each xenograft tumor formation assay (16 weeks after injection), the tumors were excised, fixed in buffered formalin, and paraffin embedded. The tissue sections were stained with hematoxylin and eosin (H&E) and immunostained for CCAR1.

Figure S11. CCAR1 is required for the assembly of AR transcription complex. LNCaP cells expressing shNS or shCCAR1 were treated with or without 10 nM DHT for 16 hr. ChIP assays using the indicated antibodies were performed as described in Figure 1C. qPCR analyses were performed using primers specific for the KLK2 (A) and FKBP5 (B) enhancers. The results are shown as percentage of input and are means ± SD (n=3).

Figure S12. CCAR1 is required for the RNA Pol II recruitment to the PSA promoter. LNCaP cells expressing shNS or shCCAR1 were treated with or without 10 nM DHT for 16 hr. ChIP assays using the indicated antibodies were performed as described in Figure 1C. qPCR analyses were performed using primers specific for the PSA promoter. The results are shown as percentage of input and are means ± SD (n=3).

Figure S13. Mapping of the CCAR1 domains involved in the interaction with AR and GATA2. (A) Schematic representation of CCAR1 structure and deletion mutants tested in GST pull-down assays. Q-rich, glutamine-rich domain; NLS, nuclear localization signal; Nudix, Nudix hydrolase domain; SAP, SAF/Acinus/PIAS domain; E/D/K-rich, glutamic acid/aspartic acid/lysine-rich domain. (B) AR interacts with the N-terminal domain of CCAR1. In vitro translated HA-tagged CCAR1 fragments were incubated with recombinant GST-AR in the presence or absence of 0.3 μM DHT. Bound proteins were analyzed by immunoblot with anti-HA antibody. (B) GATA2 interacts with the central domain of CCAR1. In vitro translated HA-tagged CCAR1 fragments were incubated with recombinant GST-GATA2. Bound proteins were analyzed by immunoblot with anti-HA antibody.

Supplementary Figure S1

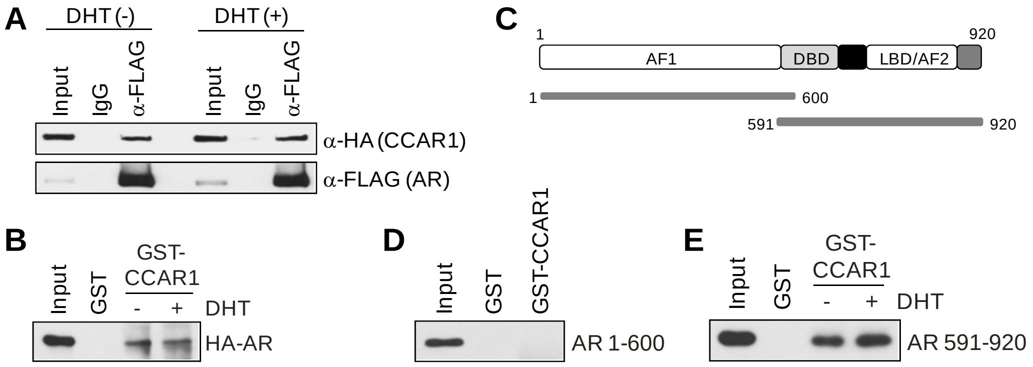

Supplementary Figure S2

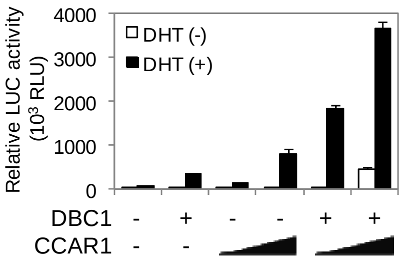

Supplementary Figure S3

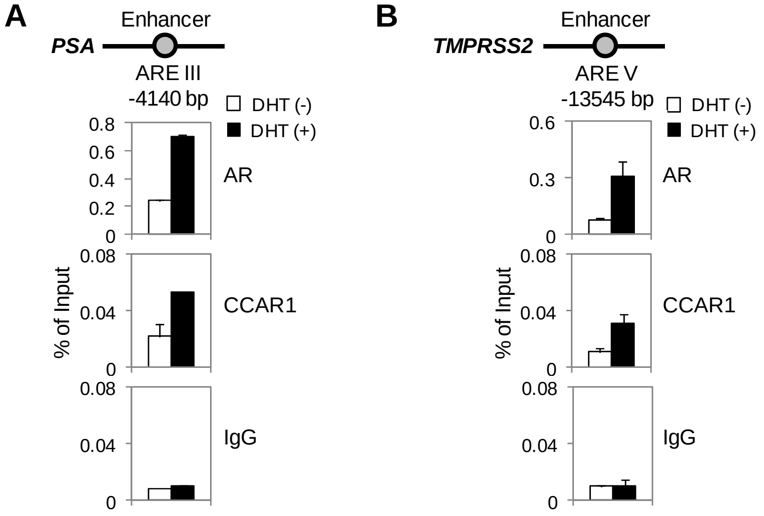

Supplementary Figure S4

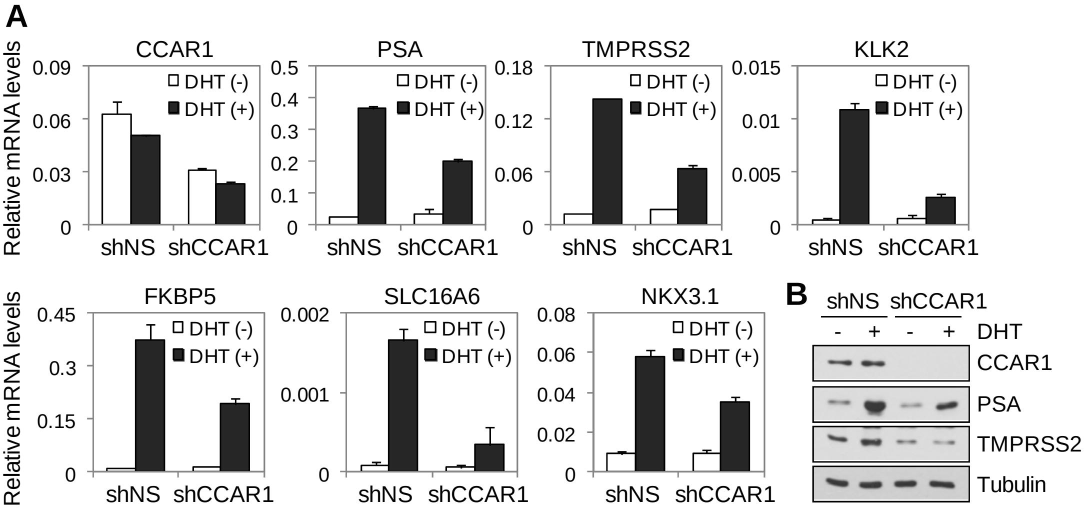

Supplementary Figure S5

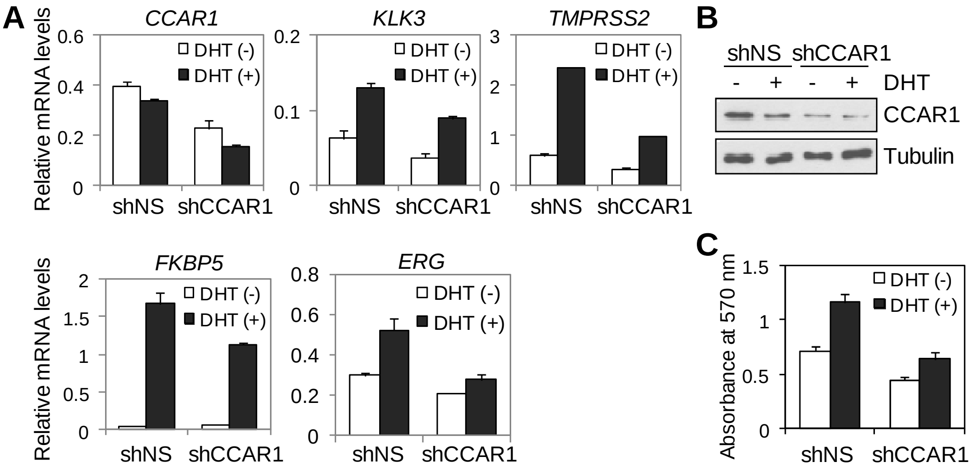

Supplementary Figure S6

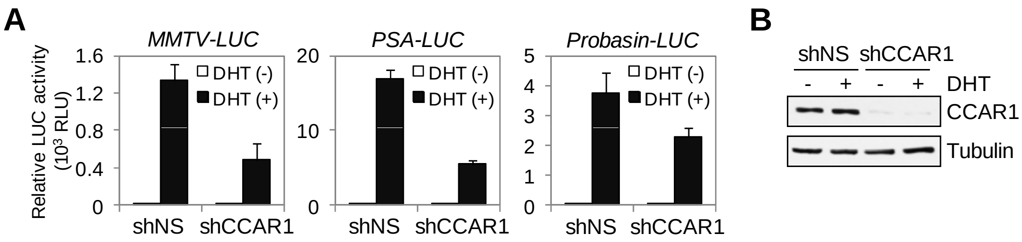

Supplementary Figure S7

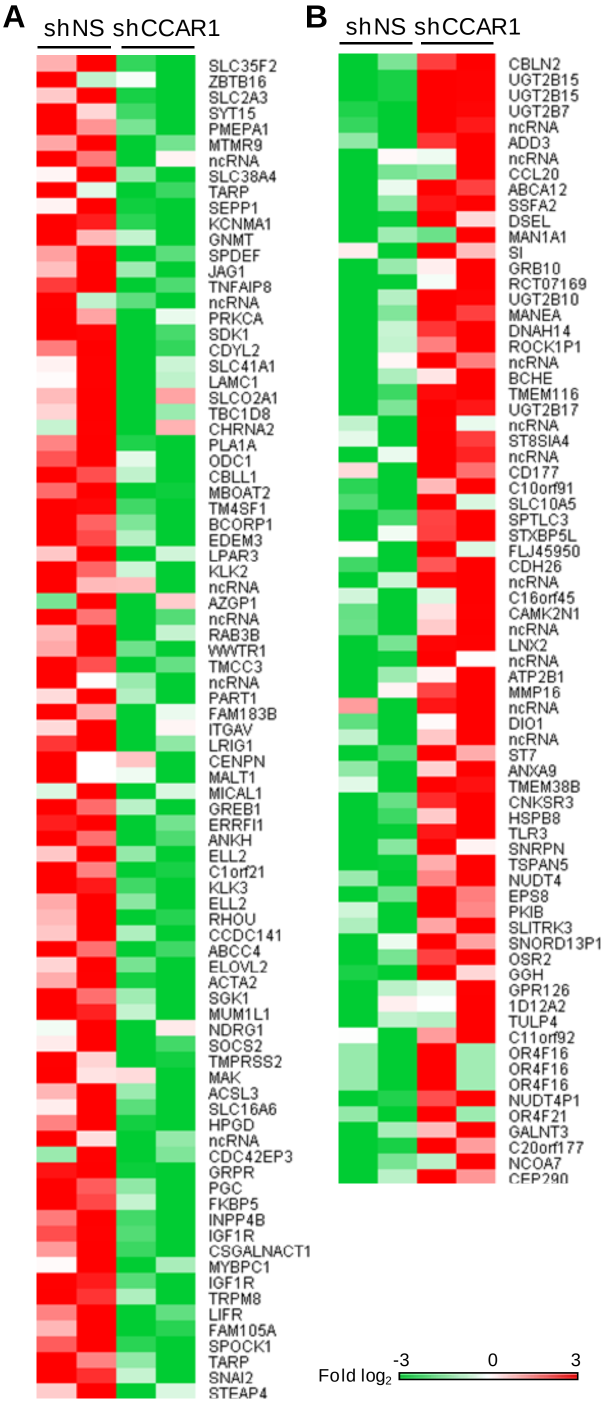

Supplementary Figure S8

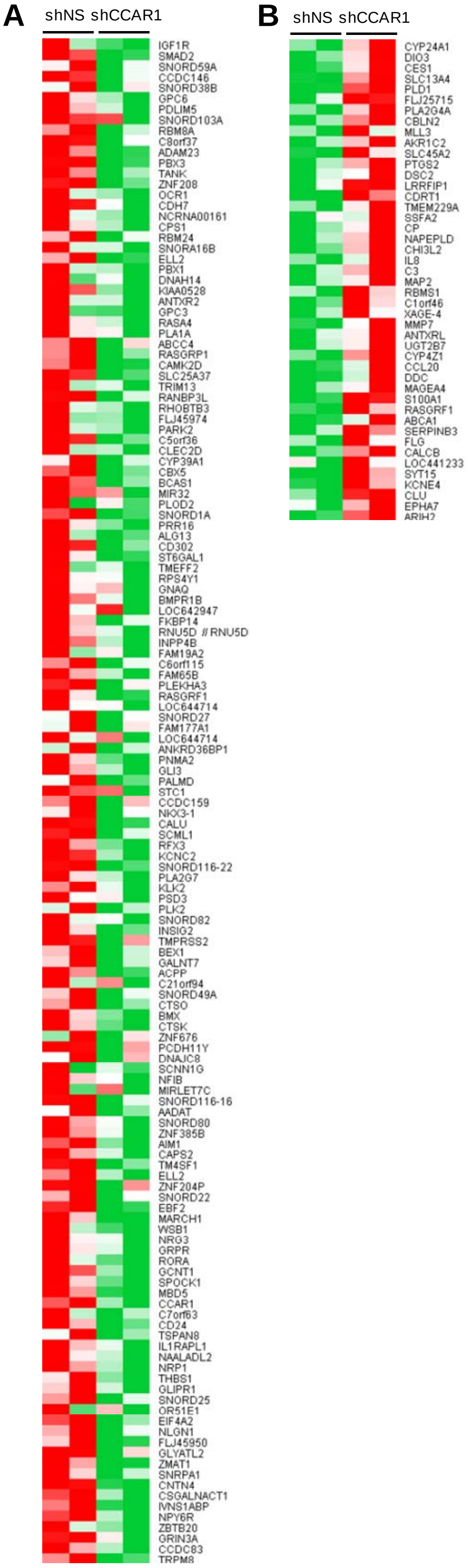

Supplementary Figure S9

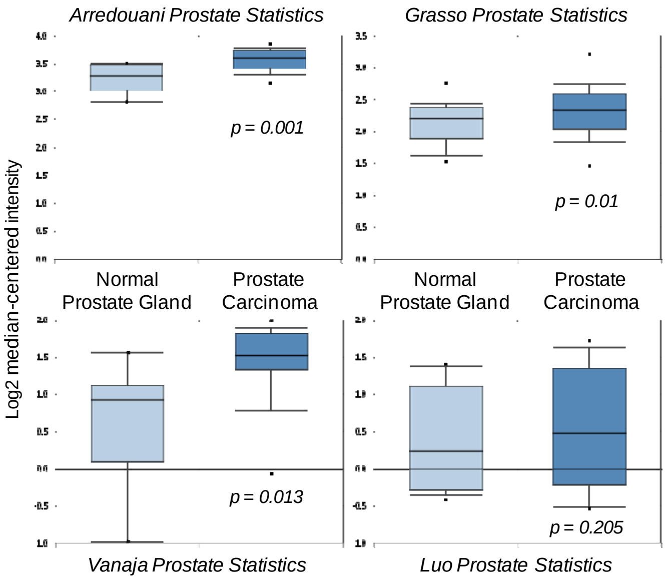

Supplementary Figure S10

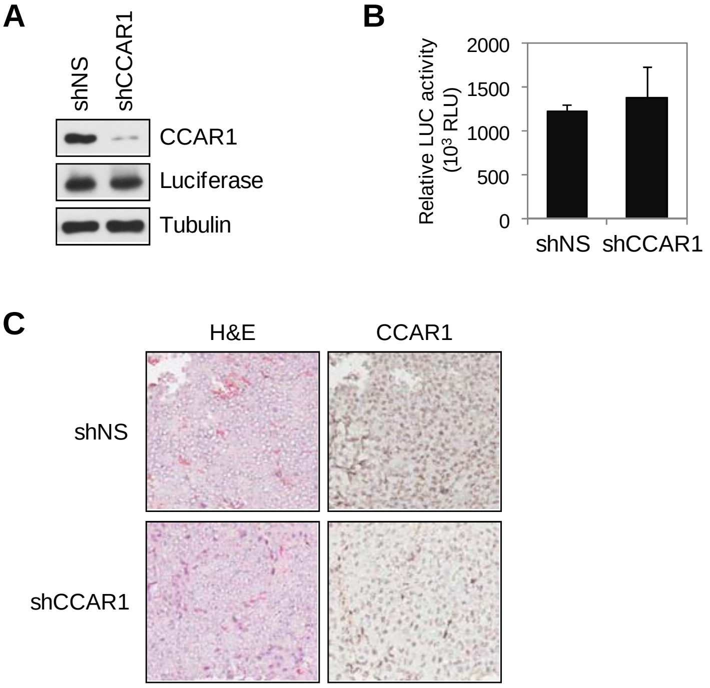

Supplementary Figure S11

A

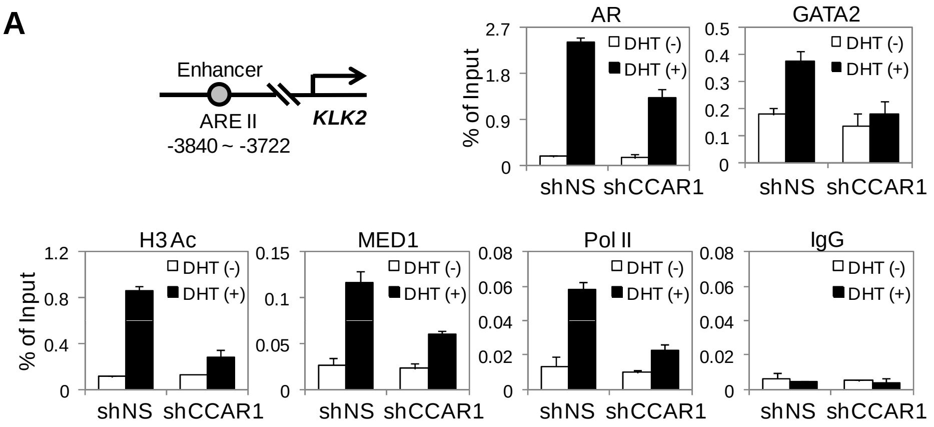

B

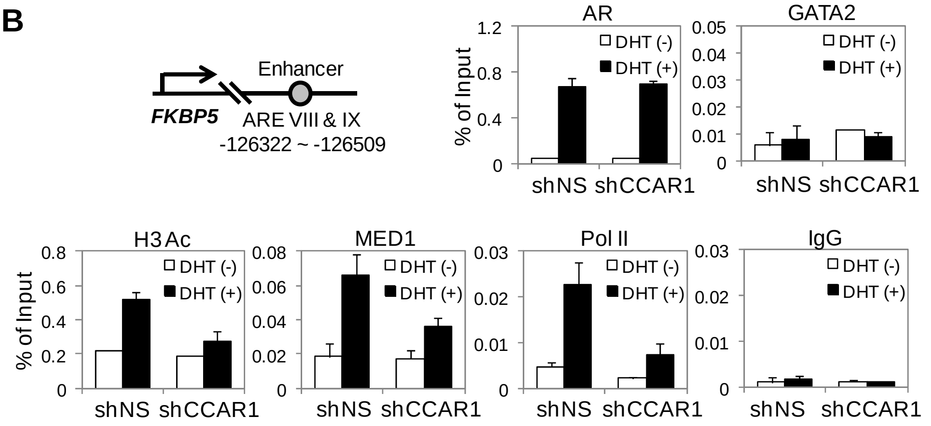

Supplementary Figure S12

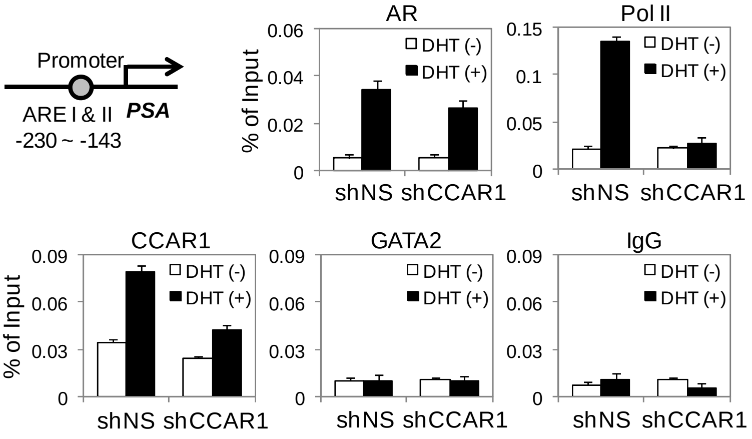

Supplementary Figure S13

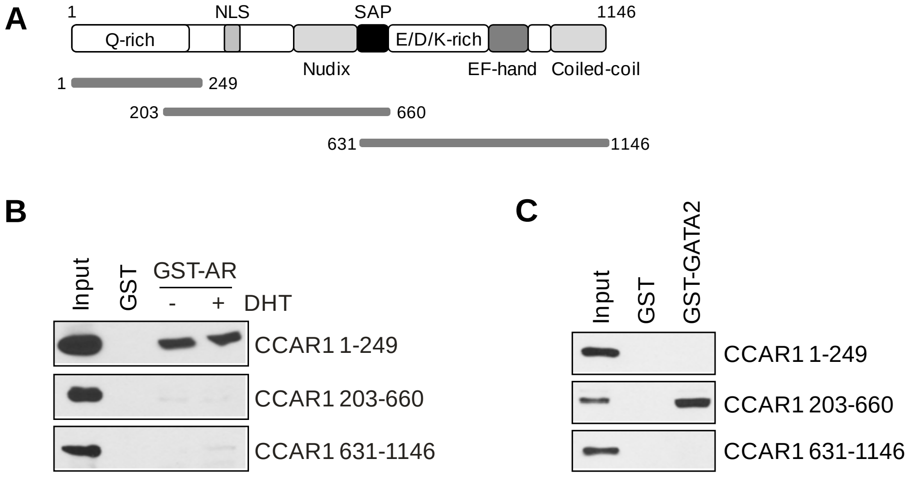

Supplement: Supplementary Data [file supp_gkt644_nar-01016-x-2013-File003.pdf]
